# The predictive value of placental growth factor combined with uterine ultrasound arterial blood flow characteristics for preeclampsia

**DOI:** 10.12669/pjms.41.2.11349

**Published:** 2025-02

**Authors:** Fengmei Xiao, Caiqiu Li, Xiaoying Wang, Jun Li

**Affiliations:** 1 Fengmei Xiao, Department of Obstetrics, Yongkang First People’s Hospital, Yongkang, Zhejiang Province 321300, P.R. China; 2 Caiqiu Li, Department of Obstetrics, Yongkang First People’s Hospital, Yongkang, Zhejiang Province 321300, P.R. China; 3 Xiaoying Wang, Department of Obstetrics, Yongkang First People’s Hospital, Yongkang, Zhejiang Province 321300, P.R. China; 4 Jun Li, Department of Obstetrics, Yongkang First People’s Hospital, Yongkang, Zhejiang Province 321300, P.R. China

**Keywords:** Artery blood flow, Placental growth factor, Preeclampsia, Pregnant women, Uterine ultrasound

## Abstract

**Objective:**

To study the predictive value of placental growth factor (PLGF) combined with uterine ultrasound artery blood flow characteristics in preeclampsia (PE).

**Methods:**

The retrospective cohort study included singleton pregnant women who admitted for prenatal examinations at the Yongkang First People’s Hospital between February 2021 to November 2023. Based on whether the pregnant women had PE and the severity of PE, they were divided into Control group, Severe PE group and Mild PE group. Levels of PLGF and ultrasound arterial blood flow parameters were compared between the groups, and the sensitivity and specificity of the above indicators were calculated.

**Results:**

This study included one hundred pregnant women with PE in the analysis, with 29 in the Severe PE group and 71 in the Mild PE group. Additionally, 100 healthy pregnant women without PE were included in the control group. Levels of PLGF, resistance index (RI), pulsatility index (PI), and systolic/diastolic blood flow ratio (S/D) were statistically different between the groups (*P*<0.05). Logistic analysis showed that PLGF, RI, PI, and S/D were risk factors for the onset of PE (*P*<0.05). Receiver operating characteristic (ROC) curve analysis showed that the combined prediction value of PLGF, RI, PI, and S/D for PE was significantly higher than the individual prediction of each indicator (*P*<0.05).

**Conclusions:**

PE was significantly associated with decreased levels of PLGF and increased RI, PI, and S/D in pregnant women. These indexes can serve as important indicators for predicting the onset of preeclampsia, especially when they are combined.

## INTRODUCTION

Preeclampsia (PE) is one high blood pressure disorder that can occur during pregnancy, with an incidence of about 5%~20%.^1^,^2^ PE patients present with clinical symptoms such as proteinuria and elevated blood pressure, which is often accompanied by systemic damage^2^ and may lead to poor maternal and neonatal outcomes.^1^–^3^ PE is caused by abnormal invasion of placental trophoblasts, insufficient reconstruction of spiral arteries in the uterine muscle layer, and endothelial damage, which leads to systemic small vessel spasms,^3^,^4^ and hypertension and proteinuria that may occur as the condition progresses.^3^–^5^ Therefore, accurate diagnostic evaluation of preeclampsia is extremely important.

Ultrasound is considered an important diagnostic tool in patients with PE as it can accurately measure arterial blood flow and detect pathological changes in arterial blood flow in the early stage of the disease.^6^ Ultrasound examination of the blood flow status of the umbilical and uterine arteries can determine the degree of vasospasm in the umbilical and uterine arteries.^6^,^7^ Studies show that placental villous and vascular histopathological lesions are more common in PE pregnancies^8^ and are associated with increased resistance to uterine artery blood flow that is detectable by Doppler assessment.^9^

Placental growth factor (PLGF) is a member of the vascular endothelial growth factor family^10^ that binds to vascular endothelial cell growth factor to form heterodimers, exerting inhibitory or promoting effects on angiogenesis.^10^,^11^ Studies have showed an important role of PLGF in predicting PE in the first trimester and its good diagnostic capacity after 20 weeks of gestation in symptomatic women.^12^,^13^ A recent review suggested that the use of PLGF testing, combined with the medical history, and maternal characteristics may significantly improve first-trimester prediction of PE.^10^ However, it has also been shown that while uterine artery Doppler velocimetry and maternal serum PlGF estimation at 20–22 weeks of gestation can strongly predict the occurrence of PE when used alone, the association with PE when the two are used in combination is not significant.^14^ Therefore, this study was conducted to further to clarify the predictive value of PLGF or uterine ultrasound artery blood flow characteristics alone or in combination for PE.

## METHODS

The study was a retrospective cohort study. Singleton pregnant women who were admitted for prenatal examinations at the Yongkang First People’s Hospital between February 2021 to November 2023 were included in the study, and the pregnancy follow-up results were recorded. Based on whether the pregnant women had PE and the severity of PE, they were divided into control group (patients without PE), severe group (patients with severe PE) and mild group (patients with mild PE).

### Ethical Approval:

The Ethics Committee at the Yongkang First People’s Hospital approved this study (No. YKSDYRMYYEC2024-LW-KS-024-01). All procedures involving human subjects adhered to the 1964 Declaration of Helsinki and its subsequent amendments or equivalent ethical standards. Given the retrospective nature of the study, informed consent was waived by the Ethical Committee of the Yongkang First People’s Hospital. All data were stored securely, and confidentiality was maintained throughout the study.

### Inclusion criteria:

- Single pregnancy.
- The clinical data was complete.

### Exclusion criteria:

- Placental implant recipients.
- People with infectious diseases or infectious diseases.
- Individuals with autoimmune, metabolic, and endocrine system diseases.
- Individuals with malignant tumors.
- Fetal chromosomal abnormalities, fetal structural abnormalities, placental abnormalities.
- Congenital uterine malformation.

### Diagnostic criteria for PE: 15,16

### A diagnosis of mild PE was made in cases where:

- Hypertension first appeared after 20 weeks of pregnancy, with systolic blood pressure ≥ 140mmHg and/or diastolic blood pressure ≥ 90mmHg.
- Proteinuria greater than or equal to 0.3g per 24 hours or randomly positive for urine protein.
- Accompanied by symptoms such as upper abdominal discomfort and headache.
- A diagnosis of severe PE was made if any of the following accompanied pregnancy:
- Uncontrolled increase in blood pressure, systolic blood pressure ≥ 160mmHg, and diastolic blood pressure ≥ 110mmHg;
- Proteinuria>3g/24h;
- Blood creatinine ≥ 90 μmol/L;
- Oligouria ≤ 500mL/24h or ≤ 25mL/h;
- Thrombocytopenia<100×10^9^/L;
- Hepatocellular necrosis with alanine aminotransferase or aspartate aminotransferase>2N;
- Persistent or severe upper abdominal pain or right rib pain;
- Chest pain, difficulty in breathing, acute pulmonary edema;
- Neurological symptoms: severe headache with poor treatment effectiveness, persistent visual and auditory problems, and deep tendon hyperreflexia.^17^

### PLGF detection:

Serum PLGF levels were tested between 11+6 and 13+6 weeks of gestation. Approximate 4 ml of fasting blood were determined by dual antibody enzyme-linked immunosorbent assay with kits purchased from mlbio (Shanghai, China).

### Ultrasound examination:

Doppler ultrasonography was performed using VOLUSON E6 and E8 ultrasound diagnostic instruments (General Electric, WI, USA) between 11+6 and 13+6 weeks of gestation with the probe frequency set to 2.5-5 MHz. Patients were assisted to lie flat, and the external iliac artery was located with the probe. The probe was then moved towards the central axis direction, using the uterine artery located 1cm away from the iliac artery at the intersection of the external iliac and uterine arteries as the sampling window. The sampling volume was set to 2mm, and an angle of ≤ 50° was maintained between the blood flow direction and the sampling line. Continuous and regular arterial waveforms of uterine arteries were detected. At least three consecutive blood flow spectrum images were obtained. Uterine artery resistance index (RI), pulsatility index (PI), and systolic/diastolic blood flow ratio (S/D) were calculated. PI= (peak systolic velocity end-diastolic blood flow velocity)/average blood flow velocity. RI= (systolic peak velocity end-diastolic blood flow velocity)/systolic peak velocity.

### Statistical analysis:

SPSS version 24.0 (IBM Corp, Armonk, NY, USA) was used for all analyses. Continuous variables were reported as mean and standard deviation (SD). One-way analysis of variance (ANOVA) was used to evaluate the statistical significance of continuous variable differences among the groups, and LSD method was used for pairwise post hoc comparison. Count data were reported as frequency (percentage) and comparison between groups was tested using Chi square test. A logistic regression model for the occurrence of PE was established, and the predictive ability of PLGF, RI, PI, and S/D indicators for the occurrence of PE was assessed using receiver operating characteristic (ROC) curves; A *p*-value less than 0.05 was considered statistically significant. All reported *p*-values were bilateral.

## RESULTS

**Fig.1:**
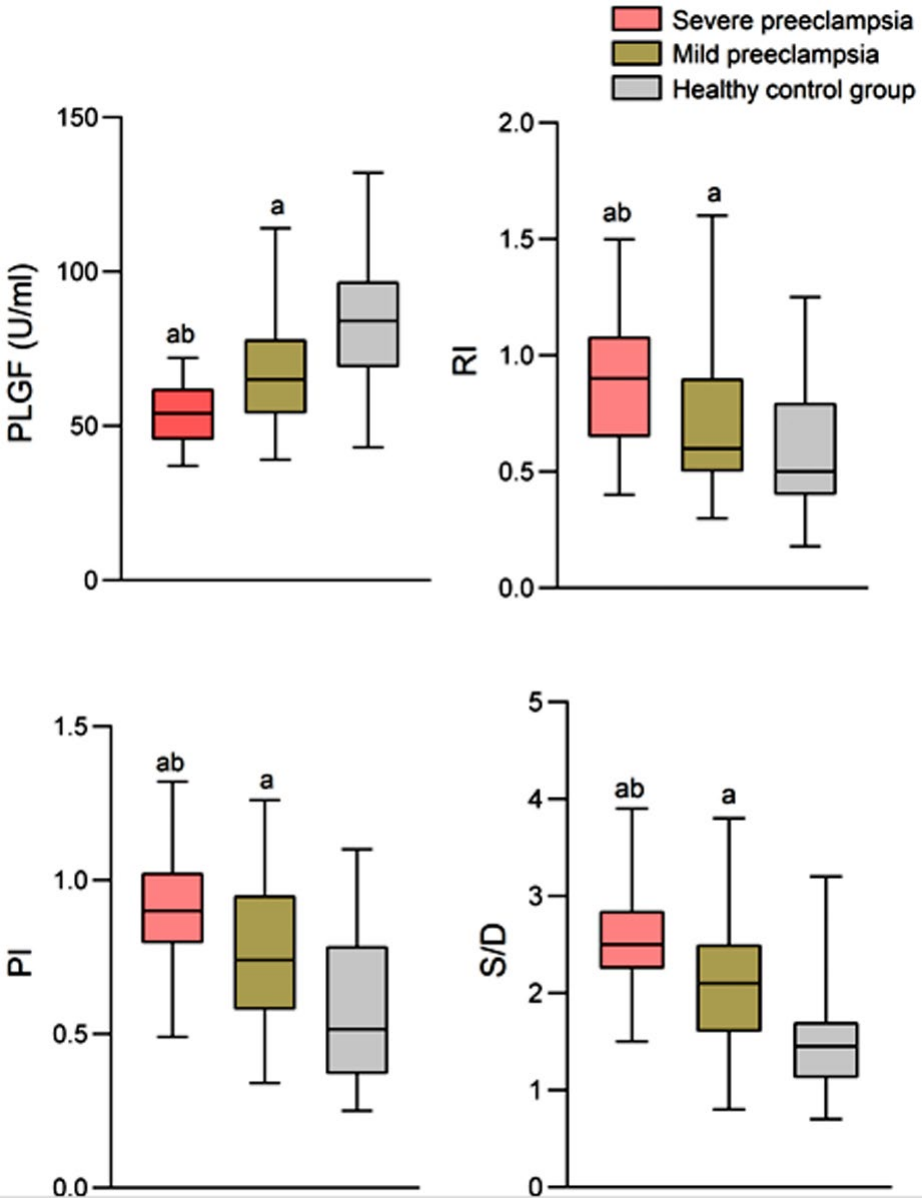
PLGF levels and uterine ultrasound arterial blood flow parameters of the study groups. PLGF: placental growth factor; RI: resistance index; PI: pulsatility index; S/D: systolic / diastolic blood flow ratio; Compared with the control group, ^a^P<0.05; Compared with the mild preeclampsia group, ^b^P<0.05.

**Fig.2:**
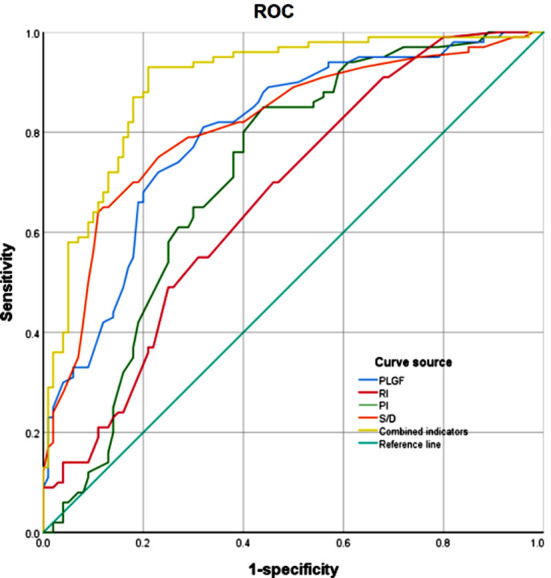
The ROC curve of each indicator predicting the occurrence of PE; PLGF: placental growth factor; RI: resistance index; PI: pulsatility index; S/D: systolic/diastolic blood flow ratio.

This study included one hundred pregnant women with PE in the analysis, of whom 29 had severe PE (Severe PE group) and 71 had mild PE (Mild PE group). Additionally, one hundred healthy pregnant women without PE were included in the control group. The age of the patients ranged from 17 to 42. As shown in Table-I, there was no significant difference in baseline data among the three groups (*P*>0.05). Diagnosis of PE was associated with significantly lower serum PLGF levels and considerably higher values of RI, PI, and S/D (p<0.05) compared to normal pregnancy Fig.1. Severe PE was associated with the lowest levels of PLGF and highest values of RI, PI, and S/D (P<0.05) Fig.1. Multivariate logistic regression analysis showed that PLGF, RI, PI, and S/D were all independent risk factors for the occurrence of PE (*P*<0.05) (Table-II). ROC curve analysis showed that PLGF, RI, PI, and S/D have a good predictive value for PE. The combined predictive value of all four parameters is higher, with a sensitivity of 93%, specificity of 79%, area under curve (AUC) of 0.897, and 95% CI: 0.853~0.94 Fig.2 and Table-III.

**Table-I:** Baseline data of the study groups.

Item	Severe preeclampsia group (n=29)	Mild preeclampsia group (n=71)	Control group (n=100)	F/χ^2^	P
Age (year)	26.83±4.75	27.30±4.39	28.54±4.36	2.541	0.081
BMI (kg/m^2^)	27.26±3.32	28.36±4.56	28.23±3.65	0.845	0.431
Multiparous women/primiparous women	5/24	13/58	26/74	1.879	0.391
Natural insemination /artificial insemination	25/3	64/7	92/8	0.250	0.882
Gravidity	1.76±1.02	1.78±1.12	1.68±0.85	0.270	0.762
Parity	1.24±0.58	1.17±0.48	1.32±0.60	1.538	0.217

BMI: body mass index.

**Table-II:** Logistic regression analysis of preeclampsia occurrence.

Index	B	S.E.	Wald χ^2^	P	OR	95%CI
PLGF	-0.051	0.011	20.843	<0.001	1.053	1.030~1.076
RI	1.427	0.677	4.437	0.035	4.166	1.104~15.715
PI	1.716	0.715	5.757	0.016	5.56	1.369~22.577
S/D	1.856	0.382	23.604	<0.001	6.4	3.026~13.532
Constant	-1.802	1.276	1.994	0.158	0.165	

***Note:*** B: partial regression system; S.E.: standard error; Wald χ^2^=(B/S.E.)^2^; OR: odds ratio; 95%CI: confidence interval of OR; PLGF: placental growth factor; RI: resistance index; PI: pulsatility index; S/D: systolic / diastolic blood flow ratio.

**Table-III:** Predictive value of PLGF and ultrasound parameters.

Index	AUC	Sensitivity (%)	Specificity (%)	Cut-off	95%CI
PLGF (U/ml)	0.799	81	68	73	0.737~0.860
RI	0.669	55	69	67.5	0.595~0.743
PI	0.716	85	56	0.57	0.644~0.789
S/D	0.815	64	89	2.05	0.755~0.875
Combined indicators	0.897	93	79	0.399	0.853~0.942

***Note:*** AUC: area under the curve; 95%CI: confidence interval of OR; PLGF: placental growth factor; RI: resistance index; PI: pulsatility index; S/D: systolic / diastolic blood flow ratio.

## DISCUSSION

Our results showed that PE is associated with significantly decreased levels of PLGF and increased RI, PI, and S/D in pregnant women. These indexes are closely related to the severity of PE and can serve as important indicators for predicting the condition’s onset. This study found that the PLGF levels in patients with PE were lower than those of healthy pregnant women, and the decrease directly correlated with the severity of the condition.

Our results further confirm previous reports.^18^-^20^ Stepan H et al.^18^ showed that PLGF can activate endothelial cells, promote their migration and proliferation, and stimulate the proliferation and invasion of trophoblasts during pregnancy, ensuring placental vascular recasting and maintaining blood supply. Moreover, the overall low expression of PLGF in PE pregnancies is even lower in severe PE cases compared to mild PE. PLGF, therefore, may play an important role in the pathogenesis and progression of preeclampsia. Stepan H et al.^19^ also showed that compared to normal pregnant women, women with PE present with considerably decreased PLGF levels, and the degree of decrease correlated with the severity of the condition. PLGF expression can greatly affect placental blood supply and angiogenesis, and monitoring its level can be used to accurately evaluate placental function in patients with PE.^20^ Tataru Copos AF et al.^21^ have pointed out that PLGF can promote placental angiogenesis and endothelial cell proliferation, support trophoblast growth, and effectively reflect placental function. Levels of PLGF in the bloodstream significantly decrease even before the onset of classical PE symptoms such as proteinuria and hypertension. Therefore, monitoring and evaluation of PLGF can be used for the early diagnosis and prediction of PE, which is consistent with our conclusions.^21^-^23^ Additionally, Baert J et al.^24^ showed that PLGF levels in patients with PE were lower than those in normal pregnant women. When the cutoff value was set at 52.635 pg/mg, PLGF levels could predict PE with an AUC of 0.869 and specificity and sensitivity reaching 71.7% and 100.0%, respectively.

The results of this study also showed that the RI, PI, and S/D of patients with preeclampsia were significantly higher than those of the control group. We detected a direct correlation between the extent of the increase and the severity of PE. Our results indicate that uterine ultrasound arterial blood flow monitoring also has high efficacy in the diagnosis and evaluation of PE. The main cause of PE is abnormal placental blood supply, leading to poor development of placental trophoblasts and insufficient infiltration of spiral arteries that negatively impacts blood supply to the fetus.^25^ The stress response caused by persistent hypoxia in the placenta can form a series of inflammatory events, leading to increased resistance of the uterine artery, contraction of the uterine smooth muscle, and further reduction of placental blood flow, forming a vicious cycle.^26^ Increased resistance can slow down the diastolic blood flow rate of the uterine artery, forming a low output and high resistance, manifested as increased RI, PI, and S/D.^27^-^29^ Zhang et al.^29^ found that the levels of RI, PI, and S/D in PE patients were higher than in normal pregnant women, and the increase in RI, PI, and S/D was greater in cases of severe PE compared to mild PE. Together with our results, these observations suggest that the abnormal increase in uterine artery resistance worsens with the progression of PE.^29^,^30^ Montaguti et al.^30^ also confirmed that the ultrasound blood flow parameters of the uterine artery in patients with PE were abnormally increased. Their study confirmed that the uterine artery blood flow resistance continuously decreased as the pregnancy progressed, mainly due to invasion of the trophoblast and transformation of the uterine spiral artery into low-resistance large blood vessels. Abnormal transformation, thus, can lead to PE and severely restrict fetal growth and development.

Our logistic regression analysis results identified PLGF, RI, PI, and S/D as important risk factors for the onset of PE. Furthermore, we showed that combining these indicators has a high application value in predicting PE. Our results further confirm the close correlation between PLGF levels and uterine artery ultrasound parameters with PE. Together, these indexes can effectively predict the incidence of PE and may be used in clinical practice to guide early prevention and control of PE.^31^,^32^

### Limitations:

Firstly, this is a single-center retrospective study with a small sample size and limited data collection. That makes our study prone to selection bias. Secondly, human or technical factors may influence PLGF and ultrasound indicators. Thirdly, the failure to group preeclampsia patients during pregnancy resulted in bias in the accuracy of PLGF, RI, PI, and S/D in predicting the occurrence of PE. Finally, the value of indicators such as PLGF, RI, PI, and S/D in predicting maternal and neonatal outcomes was not analyzed. Further higher-quality research is needed to verify our observations.

## CONCLUSION

Serum levels of PLGF significantly decreased, and RI, PI, and S/D values significantly increased in patients with PE. The changes in the PLGF levels and the ultrasound arterial blood flow parameters were closely related to the severity of PE. Serum PLGF, RI, PI, and S/D parameters, therefore, can serve as important indicators for predicting the onset of PE.

### Author’s Contributions:

**FX:** Study design, literature search and manuscript writing.

**CL, XW** and **JL:** data collection, analysis. Interpretation and critical review.

**FX:** manuscript revision and validation and is responsible for the integrity of the study.

All authors have read and approved the final manuscript.
